# T cell-mediated tumor killing sensitivity gene signature-based prognostic score for acute myeloid leukemia

**DOI:** 10.1007/s12672-024-00962-w

**Published:** 2024-04-15

**Authors:** Yiyun Pan, FangFang Xie, Wen Zeng, Hailong Chen, Zhengcong Chen, Dechang Xu, Yijian Chen

**Affiliations:** 1 Suzhou Medical College of Soochow University, Suzhou, 215123 Jiangsu People’s Republic of China; 2 Ganzhou Cancer Hospital, Gannan Medical University, No.19, Huayuan Road, Zhanggong Avenue, Ganzhou, Jiangxi People’s Republic of China; 3 Ganzhou People’s Hospital, Ganzhou, 341000 Jiangxi People’s Republic of China; 4 The First Affiliated Hospital of Gannan Medical University, No.23, Qingnian Road, Zhanggong Avenue, Ganzhou, 8105640 Jiangxi People’s Republic of China

**Keywords:** Acute Myeloid Leukaemia, Cancer immunotherapy, Prognostic score, T cell

## Abstract

**Background and Objective:**

Acute myeloid leukemia (AML) is an aggressive, heterogenous hematopoetic malignancies with poor long-term prognosis. T-cell mediated tumor killing plays a key role in tumor immunity. Here, we explored the prognostic performance and functional significance of a T-cell mediated tumor killing sensitivity gene (GSTTK)-based prognostic score (TTKPI).

**Methods:**

Publicly available transcriptomic data for AML were obtained from TCGA and NCBI-GEO. GSTTK were identified from the TISIDB database. Signature GSTTK for AML were identified by differential expression analysis, COX proportional hazards and LASSO regression analysis and a comprehensive TTKPI score was constructed. Prognostic performance of the TTKPI was examined using Kaplan–Meier survival analysis, Receiver operating curves, and nomogram analysis. Association of TTKPI with clinical phenotypes, tumor immune cell infiltration patterns, checkpoint expression patterns were analysed. Drug docking was used to identify important candidate drugs based on the TTKPI-component genes.

**Results:**

From 401 differentially expressed GSTTK in AML, 24 genes were identified as signature genes and used to construct the TTKPI score. High-TTKPI risk score predicted worse survival and good prognostic accuracy with AUC values ranging from 75 to 96%. Higher TTKPI scores were associated with older age and cancer stage, which showed improved prognostic performance when combined with TTKPI. High TTKPI was associated with lower naïve CD4 T cell and follicular helper T cell infiltrates and higher M2 macrophages/monocyte infiltration. Distinct patterns of immune checkpoint expression corresponded with TTKPI score groups. Three agents; DB11791 (Capmatinib), DB12886 (GSK-1521498) and DB14773 (Lifirafenib) were identified as candidates for AML.

**Conclusion:**

A T-cell mediated killing sensitivity gene-based prognostic score TTKPI showed good accuracy in predicting survival in AML. TTKPI corresponded to functional and immunological features of the tumor microenvironment including checkpoint expression patterns and should be investigated for precision medicine approaches.

**Supplementary Information:**

The online version contains supplementary material available at 10.1007/s12672-024-00962-w.

## Introduction

Acute myeloid leukemia (AML) refers to heterogenous hematopoetic neoplasms that are well defined and may occur spontaneously or secondarily in response to chemoradiotherapy for other malignancies [1]. Although rare, with incidence rates ranging from 0.4 to 11 cases per 100,000, the incidence of AML is anticipated to increase globally [2]. The risk factors of de-novo AML include obesity, smoking, solvent exposure and the incidence increases with age with the median age of occurrence over 60 [3]. In case of secondary AML, exposure to DNA damaging chemotherapy such as alkylating agents, platinum-based chemotherapeutics, topoisomerase inhibitors and antimetabolites and its duration increase disease risk [4]. Over the last two decades, the five year survival rate of AML has remained low at 28.7% [5]. Moreover, the survival rate in older patients has been dismally low [5]. Older, frail patients may receive palliative care [6]. Treatment toxicity and relapse have been common complications in AML, with relapse rates up to 50% [7]. The standard treatment of AML has involved chemotherapy and allogeneic hematopoietic stem cell transplantation until recently [5, 8]. In the last few years, tremendous advances have been made in the treatment of AML, with the advent of clinically approved targeted therapeutics including FLT3, IDH1, and IDH2 inhibitors [9, 10]. Next generation hypomethylating agents (HMA) that overcome the issues of resistance and HMA combination agents are increasingly applied in AML management [11].

In the analysis based on cytomorphology, cytogenetics, molecular genetics, and immunophenotyping, the following seven subtypes of TCGA_LAML (The Cancer Genome Atlas_Acute Myeloid Leukemia) were distinguished and subjected to mutation profiling: AML with minimal differentiation (M0), AML without maturation (M1), AML with maturation (M2), acute myelomonocytic leukemia (M4), acute monoblastic leukemia (M5a), acute monocytic leukemia (M5b), and acute erythroid leukemia (M6). The most frequently mutated genes in these subtypes were identified as RUNX1 in M0 (43%), NPM1 in M1 (42%), DNMT3A in M2 (26%), NPM1 in M4 (57%), M5a (49%), and M5b (70%), and TP53 in M6 (36%) [12]. This classification and mutation analysis highlight the heterogeneity within acute myeloid leukemia (AML) and emphasize the significance of genetic mutations in guiding diagnosis, prognosis, and the development of treatment strategies [12]. Such defects drive variable oncogenesis, exemplified by NPM1-mutated (25–35% cases) and DNMT3A-mutated (26% cases) subtypes [12]. NPM1-mutated AMLs frequently harbor exon-12 mutations causing cytoplasmic dislocation and oncoprotein interactions activating proliferation signaling via the RAS/MAPK cascade. DNMT3A-mutated AMLs carry catalytic domain mutations causing DNA hypomethylation, developmental gene activation, and blocked myeloid differentiation.

Over the last two decades, the five-year survival rate of AML patients has remained at a low level of 28.7% [5]. The survival rate in elderly patients is even more alarmingly low [5], and frail elderly patients often can only receive palliative care [6]. Treatment-related toxicity and relapse have been common complications of AML, with relapse rates up to 50% [7]. Until recently, the standard treatment regimen for AML remained chemotherapy and allogeneic hematopoietic stem cell transplantation [5, 8]. However, in the past few years, tremendous progress has been made in the treatment of AML, with multiple targeted drugs for mutations such as FLT3, IDH1, and IDH2 being clinically approved [9, 10]. In 2018, the FDA approved Ivosidenib and Enasidenib, two targeted inhibitors for IDH1 and IDH2 mutations, respectively. Clinical trials have shown that these drugs can significantly improve the prognosis of patients with relapsed or refractory AML [13]. In a randomized, double-blind, placebo-controlled phase III clinical trial, Ivosidenib extended the median overall survival of patients with relapsed/refractory IDH1-mutated AML from 4.3 months to 8.8 months and increased the objective response rate from 13 to 42% [14]. A phase II clinical trial of Enasidenib in patients with relapsed/refractory IDH2-mutated AML showed that 19.6% of patients achieved complete remission, with a median overall survival of 8.8 months [15]. These breakthrough advancements have brought new hope to patients with relapsed/refractory AML. Moreover, next-generation hypomethylating agents such as Guadecitabine and CC-486 have overcome the resistance issues of traditional hypomethylating agents through optimized dosing strategies. Guadecitabine combined with standard clinical treatment regimens has shown good efficacy and tolerability in elderly AML patients [16]. The oral hypomethylating drug CC-486 can reduce the risk of relapse and prolong survival in AML maintenance therapy [17]. The combination of hypomethylating agents with other novel drugs (such as the Bcl-2 inhibitor Venetoclax) has further expanded their clinical benefit population [11]. These treatment modalities offer new options for AML patients and rewrite the efficacy of traditionally poor prognostic populations. However, the efficacy of targeted drugs and next-generation hypomethylating agents in newly diagnosed and relapsed/refractory AML still varies, and their long-term survival benefits await further confirmation by large-scale prospective studies. Molecular marker-guided precision medication and novel immunotherapy strategies are current research hotspots.

The tumor microenvironment in AML is immunosuppressive and marked by T cell hypofunctions [11, 18]. These include T-cell senescence and T-cell exhaustion due to apoptosis [19]. T cell function suppression in AML correlates with patient survival [20] and T-cell deregulation is implicated in immune escape AML relapse after allogeneic hematopoietic cell transplantation [21]. Therefore, immunotherapy, that harnesses the tumor killing potential of T-cells in AML, is an active research area in AML. Antibody-based approaches and chimeric antigen receptor T-cells (CAR T-cells) have shown early efficacy [22, 23]. T-cell based immunotherapy approaches under development include specific dual antigen targeting antibodies against CD123, CD33, CD13, CL-1 and T-cell immune checkpoint inhibitors targeting PD-1, PDL-1, CTLA4, TIMP3 and other emerging targets [24]. T cell response in the tumor microenvironment can be reactivated by targeting immune checkpoints [25]. Expression patterns of immune checkpoints in AML are associated with mutational status and overall survival, and bone marrow T cell populations in AML exhibit variable immune checkpoint inhibitor expression patterns [26, 27]. However, resistance to T-cell targeting immunotherapies in AML owing to variability in T cell phenotypes, remains a challenge [28]. T-cell transcriptional signatures and markers that correspond to immunotherapy response and resistance in AML is an area of intensive research focus [29]. In the present study, we constructed a signature-based T cell mediated tumor killing sensitivity genes (GSTTK) in AML and assessed its predictive performance for immunotherapy efficacy and prognosis. Further, we applied molecular docking techniques to identify novel potential agents targeting T cell-mediated tumor killing sensitivity genes in AML.

## Materials and methods

### Data download and pre-processing

The TCGA-LAML dataset was obtained from XENA (https://xenabrowser.net/datapages/) and the GTEx dataset (https://xenabrowser.net/datapages/?cohort=GTEX) [30] was downloaded as control. The two datasets were de-batched and combined into the training set data. GSE71014 [31–33] and GSE37642 [34–37] datasets were downloaded from GEO (https://www.ncbi.nlm.nih.gov/geo/) and utilized as the test dataset. The sample information is summarized in Table 1. Table 2 depicts the clinical characteristics and subtypes of the TCGA-LAML data. Next, 641 T cell-mediated tumor killing sensitivity-related genes (GSTTK) were downloaded from the TISIDB database (http://cis.hku.hk/TISIDB/), of which 588 genes were present in the training set.

**Table 1:** Datasets and sample information

Database	Tumor	Normal	Total
TCGA-LAML	132	70 (GTEx)	202
GSE71014	104	0	104
GSE37642	136	0	136

**Table 2:** Clinical characteristics and subtypes of the TCGA-LAML samples

	High (N = 66)	Low (N = 65)	Overall (N = 131)
Age			
< 60	31 (47.0%)	45 (69.2%)	76 (58.0%)
> = 60	35 (53.0%)	20 (30.8%)	55 (42.0%)
Gender			
Female	26 (39.4%)	34 (52.3%)	60 (45.8%)
Male	40 (60.6%)	31 (47.7%)	71 (54.2%)
History_of_neoadjuvant_treatment			
No	46 (69.7%)	54 (83.1%)	100 (76.3%)
Yes	20 (30.3%)	11 (16.9%)	31 (23.7%)
Subtype			
Subtype.1	8 (12.1%)	0 (0%)	8 (6.1%)
Subtype.2	9 (13.6%)	5 (7.7%)	14 (10.7%)
Subtype.3	0 (0%)	16 (24.6%)	16 (12.2%)
Subtype.4	12 (18.2%)	16 (24.6%)	28 (21.4%)
Subtype.5	13 (19.7%)	13(20.0%)	26 (19.8%)
Subtype.6	17 (25.8%)	9 (13.8%)	26 (19.8%)
Subtype.7	7 (10.6%)	6 (9.2%)	13 (9.9%)

The selection of these two GEO datasets were based on specific inclusion criteria. We searched the GEO database for datasets related to acute myeloid leukemia (AML) that met the following requirements: (1) The dataset contained gene expression data from AML patient samples, with the experimental type being “Expression profiling by array” to ensure consistency in the data generation method and comparability between datasets. (2) The dataset included clinical information, particularly overall survival data, for the AML patients. (3) The dataset had a sufficient sample size (n > 50) to ensure statistical power and reliability of the analyses. (4) The dataset was generated using a well-established gene expression profiling platform, such as Affymetrix or Illumina, to ensure data quality and comparability with other datasets.

Prior to the main analysis, the raw gene expression data underwent a comprehensive preprocessing pipeline to ensure data quality and reduce technical noise. Firstly, samples with a high percentage of missing values (> 10%) or low overall expression levels (bottom 10th percentile) were excluded, and genes with a high percentage of missing values (> 20%) across all samples were removed. Any remaining missing values were imputed using the k-nearest neighbor (KNN) algorithm, with the number of nearest neighbors set to 10, using the ‘impute.knn’ function from the 'impute' package in R. To reduce skewness and improve normality, the raw expression values were log2 transformed. To minimize the impact of potential batch effects arising from different data sources or experimental conditions, we applied the ComBat method from the 'sva' package [38] in R, which empirically estimates and removes batch effects by adjusting the gene expression values based on a linear model that incorporates batch information. The version numbers of all R packages used in this study are provided in Additional File 1.

### Differential expression analysis

Differentially expressed genes among the 588 GSTTK in the training set data were identified using the Wilcox.Test function [39] from the ‘stats’ package in R (version 3.6.2) [40]. The ‘stats’ package [40] provides a collection of statistical functions, including the Wilcoxon rank-sum test, which is suitable for comparing gene expression between two groups (AML samples vs. control samples) when the data is not normally distributed. The differentially expressed genes (DEGs) were selected based on the following criteria: p-value < 0.05 and |log2FC|> 1, where FC represents the fold change in gene expression between the AML and control groups.

### Enrichment analysis

GO and KEGG enrichment analysis of the differentially expressed GSTKK was performed using the clusterProfiler package [41] in R (version 3.6.2), with parameters p adjustment method = BH’, p value cutoff = 0.05, q value cutoff = 0.2.

### Protein–protein interaction (PPI) network

The STRING database [42] was used protein–protein interaction (PPI) pair-based network construction using the differentially expressed GSTKK. The screening criteria included a minimum required interaction score = 0.7, with the removal of non-connected genes. The network was mapped using Cytoscape (v3.8.0) [43]. The network topology analysis on the PPI network using the NetworkAnalyzer tool in Cytoscape [43]. The obtained results were analyzed in descending order according to the degree, and nodes with a degree ≥ 30 were extracted.

### Construction of a T cell-mediated tumor killing sensitivity gene-based prognostic score index: TTKPI

The ‘coxph’ function [44] in the ‘surv’ R package [45] was used to perform one-way COX regression analysis to predict survival outcome, with the 401 differentially expressed genes as predictor features, screened at a p value < 0.05. 57 genes with significant prognostic effects were obtained. Next, the ‘glmnet’ function [46] in the ‘glmnet’ R package [46] was used to perform LASSO regression analysis for the significant genes obtained from the step mentioned above, with parameters set at alpha = 1, nlamba = 100, and a significance level of p < 0.05. LASSO regression analysis was employed to further refine the gene selection and obtain a more stable and interpretable prognostic model. By imposing an L1 penalty on the regression coefficients, LASSO selects a subset of the most relevant genes and reduces overfitting, leading to a more generalizable prognostic model. In our study, LASSO regression was applied to the genes that were significant in the univariate Cox analysis, aiming to identify a parsimonious set of genes with the strongest prognostic value for constructing the TTKPI risk score.

The formula used for calculating the TTKPI risk score was adapted from similar risk score models employed in previous cancer prognostic studies [47–49]. The general concept of constructing a weighted sum of gene expression values to create a prognostic score has been widely used in various cancer types. The resulting genes selected by LASSO were used to construct a risk score for tumor killing (TTKPI). The scores were computed using the equation:

$${\mathrm{Risk score}}_{{\text{i}}} = \sum_{{\text{j}}=1}^{{\text{n}}}{{\text{C}}}_{{\text{j}}}*{{\text{exp}}}_{{\text{ij}}}$$

This equation calculates the risk score value of the ith sample, where C_j is the regression coefficient of the jth prognostic factor in the model, and exp_ij denotes the expression of the jth prognostic factor for the ith sample. The risk score model for predicting sample survival was established by weighting the expression of the significant genes with LASSO regression coefficients (exp represents gene expression level, C represents lasso regression coefficient).

### Survival analysis with TTKPI scores

Samples were grouped into high and low TTKPI risk score groups based on the median level. Survival curves were generated using the Kaplan–Meier method to predict overall survival. Univariate and multifactorial Cox proportional hazard analyses were performed to determine the prognostic value of the risk scores in combination with other clinical characteristics. Receiver operating curves (ROCs) were used to estimate the predictive performance of the TTKPI risk model for 1-, 3-, and 5-year survival, correlation analyses were performed using the ‘cor.test’ function, and Fisher’s exact tests were performed, where a p value < 0.05 was considered a statistically significant difference.

### Methylation analysis of the 24 signature genes

To investigate the methylation status of the 24 signature genes in AML patients, methylation data (Illumina Human Methylation 450) for AML was downloaded from The Cancer Genome Atlas (TCGA) database. Samples labeled as “Primary Blood Derived Cancer–Peripheral Blood” and “Blood Derived Normal” were selected for the analysis. Using the ChAMP package [50] in R, samples with NA expression values were removed, and normalization and differential expression analysis were performed. After sample selection, a total of 29 samples were obtained, including 21 disease samples and 8 normal samples. From the normalization and differential expression analysis results, the methylation site expression information corresponding to the 24 signature genes was extracted. Out of the 24 genes, only 20 genes had corresponding methylation expression values. A heatmap was then generated to visualize the methylation patterns of these genes across the AML and normal samples. To identify differentially methylated sites, a threshold of adjusted P-value < 0.05 and |logFC|≥ 0.2 was applied in the differential expression analysis. The results were visualized using a volcano plot, which highlights the significantly differentially methylated sites between the AML and normal groups.

### Mutation analysis of the signature genes

To investigate the mutation status and frequency of the 24 signature genes in AML, mutation data for AML was downloaded from TCGA database. The downloaded mutation data included information on the genomic location, mutation type (e.g., missense, nonsense, frameshift), and variant allele frequency (VAF) for each identified mutation. Using custom scripts in R, the mutation data was processed and filtered to extract the mutation sites and frequencies specifically within the 24 signature genes. The mutation information for each gene was then summarized, including the total number of mutations, the specific mutation sites, and the corresponding VAFs. To visualize the mutation landscape of the signature genes in AML, a mutation waterfall plot was generated using the 'GenVisR' package [51] in R. The waterfall plot displays the mutation status and frequency of the signature genes across the AML samples, with each row representing a specific gene and each column representing an individual sample.

### Association of TTKPI scores with clinical phenotype in AML

We further analysed the TCGA dataset by extracting the clinical characteristics of the samples including age, gender, adjuvant treatment status, and cancer staging. The TTKPI scores were compared between clinical subgroups. Univariate and multivariate Cox regression analyses (at a threshold of the p value < 0.05) were performed to test the predictive performance of TTKPI scores and clinical characteristics of the TCGA dataset. The results were plotted as forest plots. The TTKPI risk grouping was combined with the clinical characteristics that were significant in the univariate and multivariate Cox proportional hazards analysis, and the nomogram function in the R package ‘rms’ [52] was applied to construct a column line plot for nomogram analysis. Calibration curves were plotted using the calibrate function [53] and decision curve analysis (DCA) was performed by plotting decision curves.

### Tumor immune cell infiltration analysis and immune checkpoint expression in TTKPI groups

Tumor infiltration analysis was based on the gene expression data of TCGA, and the proportion of tumor infiltrating immune cells (22 immune cells) in a sample was determined using CIBERSORT in R. The analysis was performed using the default parameters infiltration scores for 22 immune cells were obtained. The differences in immune checkpoint expression levels between high and low TTKPI groups were analysed.

### Molecular docking association with TTKPI to predict potential therapeutic agents.

Corresponding compound structures were downloaded from the DrugBank database and screened according to Lipinski’s rule (hydrogen bond acceptor <  = 10, hydrogen bond donor <  = 5, rotatable bond <  = 10, logarithmic value of lipid-water partition coefficient <  = 5, molecular weight of 180–480, polar surface area <  = 140). The spatial structure information of key gene-encoded proteins was searched in the PDB database and the corresponding PDB files were downloaded. The approximate docking box range was determined based on the ligand information therein, and other relevant parameters of autodock-vina were set and used to dock small molecule compounds. Interaction force analysis was performed using Pymol [54] and Ligplus [55].

## Results

### Aberrant expression and function of GSTTKs in AML

**Fig. 1:**
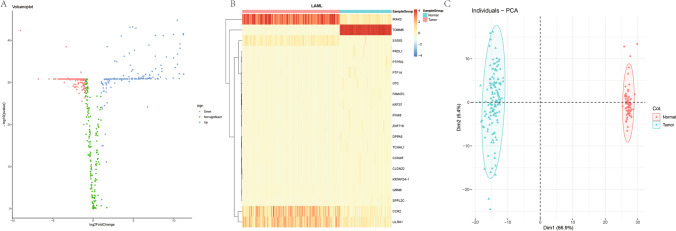
**A**. Differential gene volcano map, **B**. Differential gene heat map (TOP20), **C**. PCA analysis map

Differential analysis of 588 GSTTKs in the test set data by rank sum test (screening criteria, p < 0.05, |log2FC|> = 1) yielded 401 DEG, where 171 were up-regulated and 230 were down-regulated. A volcano plot (Fig. 1A), differential gene expression heat map (screening the top 20 genes according to |log2FC| (Fig. 1B) and a PCA analysis plot (Fig. 1C) illustrating the GSTTKs that distinguish AML samples from controls (the control samples were de-batched GTEx samples) are presented.

**Fig. 2:**
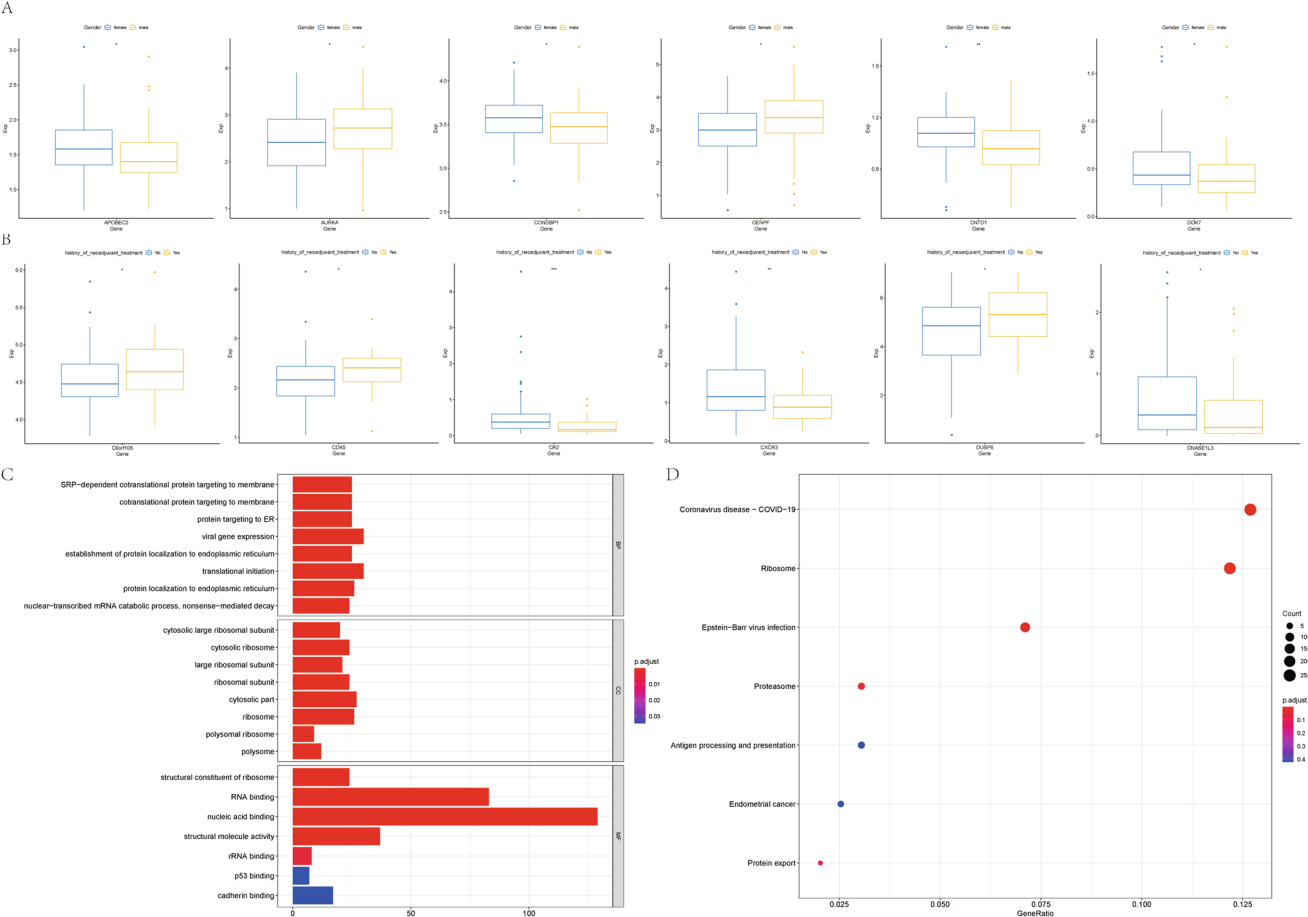
**A**. 6 differentially expressed GSTTK associated with sex, **B**. 6 differentially expressed GSTTK associated with whether adjuvant therapy was performed, **C**. GO enrichment analysis, **D**. KEGG enrichment analysis

The relationship between the 401 differential GSTKK and clinical characteristics (gender, history of adjuvant therapy or not) was analysed separately, and six genes were selected for visualization (Fig. 2A, B).

The results of GO and KEGG functional enrichment analyses were based on protein-related pathways, immune-related pathways including antigen–antibody-related, T-cell-related, and leukocyte-related pathways, including antigen processing and presentation of exogenous peptide. The results of GO enrichment analysis included more than 20 immune-related pathways including antigen processing and presentation of exogenous peptide via MHC class I, T cell extravasation, interleukin-1-mediated signalling pathway. The results of KEGG enrichment analysis included the Antigen processing and presentation pathway. The results are shown in Fig. 2C, D.

**Fig. 3:**
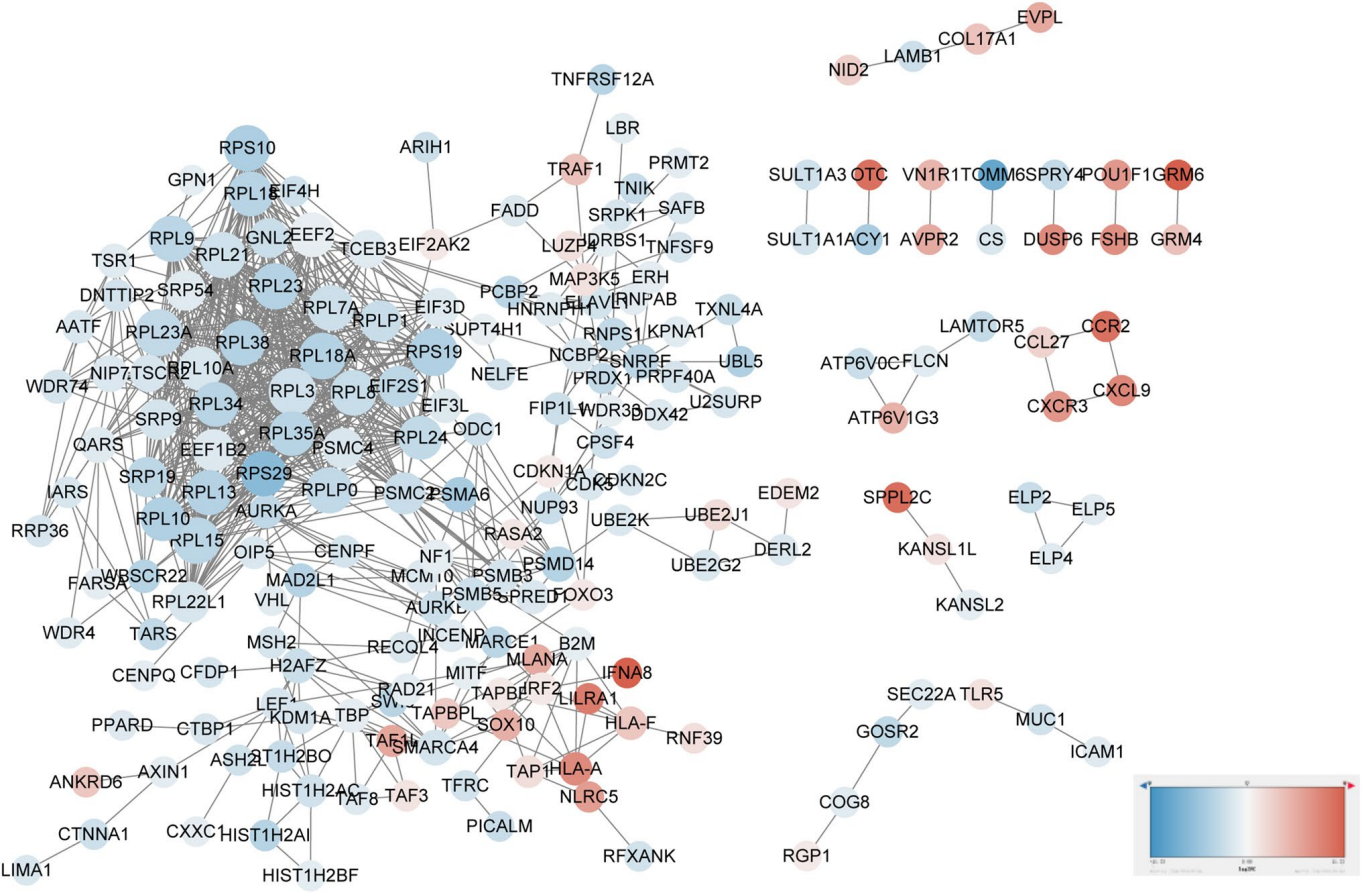
Gene interaction network for the differentially expressed GSTTK constructed using Cytoscape. Node size corresponds to the connectivity and the color corresponds to the log2FC value

The gene interaction network showed genes with high connectivity, included RPS19, RPL3, RPL23A, and the log2FC values were negative (Fig. 3). Based on the topological property analysis of the protein–protein interaction (PPI) network using Cytoscape’s NetworkAnalyzer, nodes with a degree ≥ 30 were extracted and presented in Table 3. Table 3 revealed that RPS19 had the highest degree [38] in the network, indicating its extensive interactions with other genes and potential influence on biological functions. Other nodes with high degrees included RPL3 [36], RPL23A [36], RPL18A [35], RPL15 [35], RPLP0 [34], RPL21 [34], RPL23 [34], RPS10 [34], and RPL9 [34]. These genes, predominantly belonging to the ribosomal protein family, exhibited high connectivity within the network.

**Table 3:** Topological properties of nodes with degree ≥ 30 in the protein–protein interaction network

Name	log2FC	Degree	AverageShortest PathLength	Betweenness centrality	Closeness centrality	Clustering coefficient	Neighborhood connectivity	Topological coefficient
RPS19	− 4.35379	38	3.084967	0.041556	0.324153	0.627312	26.05263	0.420204
RPL3	− 2.44812	36	3.189542	0.013518	0.313525	0.695238	27.33333	0.479532
RPL23A	− 3.33418	36	3.104575	0.018108	0.322105	0.709524	27.52778	0.451275
RPL18A	− 4.17283	35	3.111111	0.013969	0.321429	0.744538	28.11429	0.46089
RPL15	− 3.7385	35	3.117647	0.01432	0.320755	0.72605	27.62857	0.460476
RPLP0	− 3.32946	34	3	0.162284	0.333333	0.723708	27.94118	0.443044
RPL21	− 2.48237	34	3.124183	0.010671	0.320084	0.777184	28.52941	0.47549
RPL23	− 4.11393	34	3.124183	0.010671	0.320084	0.777184	28.52941	0.47549
RPS10	− 4.63799	34	3.20915	0.011766	0.311609	0.73262	27.79412	0.496324
RPL9	− 3.70758	34	3.202614	0.021696	0.312245	0.737968	27.94118	0.498424
RPL8	− 2.96921	33	3.20915	0.005509	0.311609	0.808712	29.0303	0.509304
RPL7A	− 2.70929	33	3.202614	0.006252	0.312245	0.795455	28.81818	0.496865
RPL13	− 4.10429	32	3.267974	0.008831	0.306	0.802419	28.78125	0.513951
RPL38	− 4.20531	32	3.189542	0.009478	0.313525	0.794355	28.8125	0.480208
RPS29	− 6.79452	32	3.366013	0.006854	0.297087	0.768145	28.125	0.551471
EEF2	− 1.00853	31	3.084967	0.168275	0.324153	0.709677	27.58065	0.466922
RPL35A	− 3.91115	31	3.352941	0.005002	0.298246	0.832258	29.29032	0.542413
RPL34	− 3.88897	31	3.281046	0.006612	0.304781	0.84086	29.25806	0.531965
RPL18	− 3.95552	31	3.477124	0.0029	0.287594	0.827957	29.16129	0.57179
RPL10A	− 1.91508	30	3.163399	0.00693	0.316116	0.832184	29.7	0.512069
RPL10	− 4.36184	30	3.248366	0.002625	0.307847	0.878161	30.16667	0.558642

The table includes the gene name, log2FC (log2 fold change), degree, average shortest path length, betweenness centrality, closeness centrality, clustering coefficient, neighborhood connectivity, and topological coefficient for each node. Nodes are sorted in descending order based on their degree

### Construction and validation of TTKPI

**Fig. 4:**
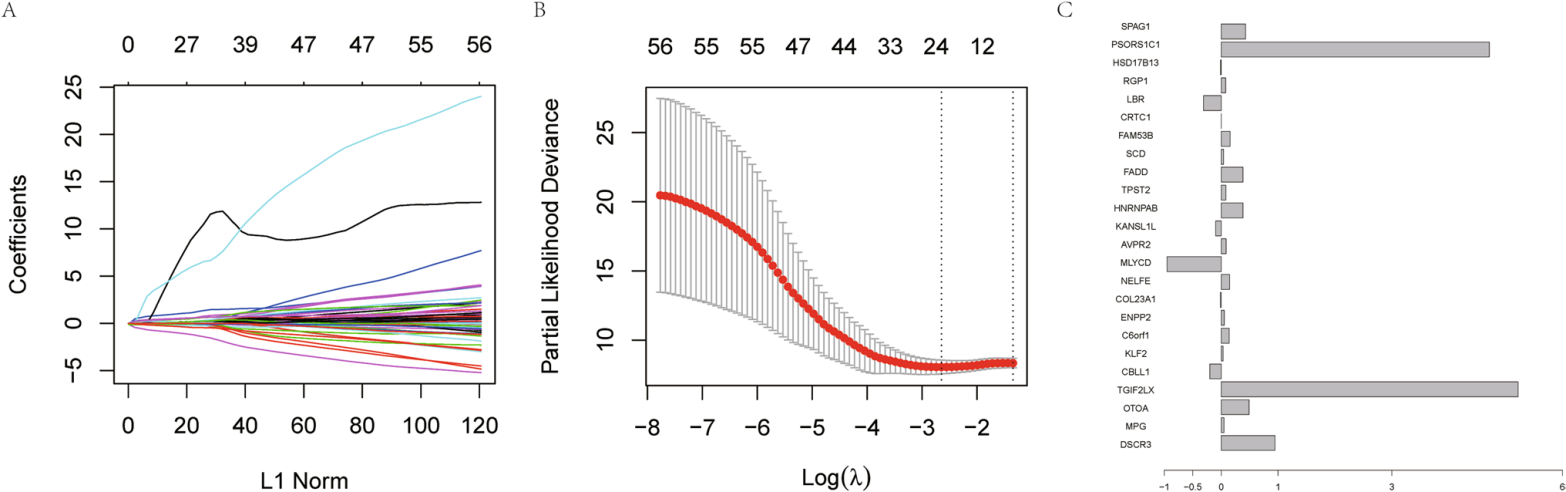
**A**. LASSO analysis to screen redundant genes., **B**. LASSO analysis and distribution of lambda values. **C**. coefficients statistics of 24 genes

**Fig. 5:**
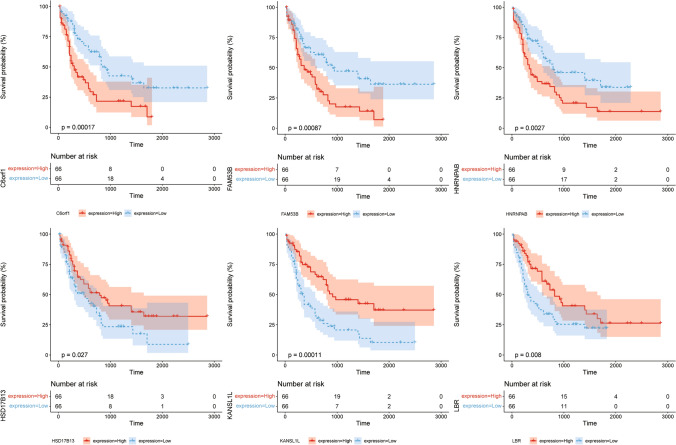
Kaplan Meier survival curves for 6 of the 24 GSTKK genes selected by LASSO regression analysis

A one-way cox regression analysis of TCGA dataset with the 401 differential GSTTK was performed and 57 genes with significant prognostic value were further screened using LASSO regression. 24 key prognostic genes were determined using LASSO including GSTKK; DSCR3, MPG, OTOA, TGIF2LX, CBLL1, KLF2, C6orf1, ENPP2, COL23A1, NELFE, MLYCD, AVPR2, KANSL1L, HNRNPAB, TPST2, FADD, SCD, FAM53B, CRTC1, LBR, RGP1, HSD17B13, PSORS1C1, SPAG1 (Fig. 4A–C), and Kaplan–Meier survival curves were plotted for these 24 genes (see Additional file 2: Appendix for details), and the survival curves for six of these are shown (Fig. 5).

**Fig. 6:**
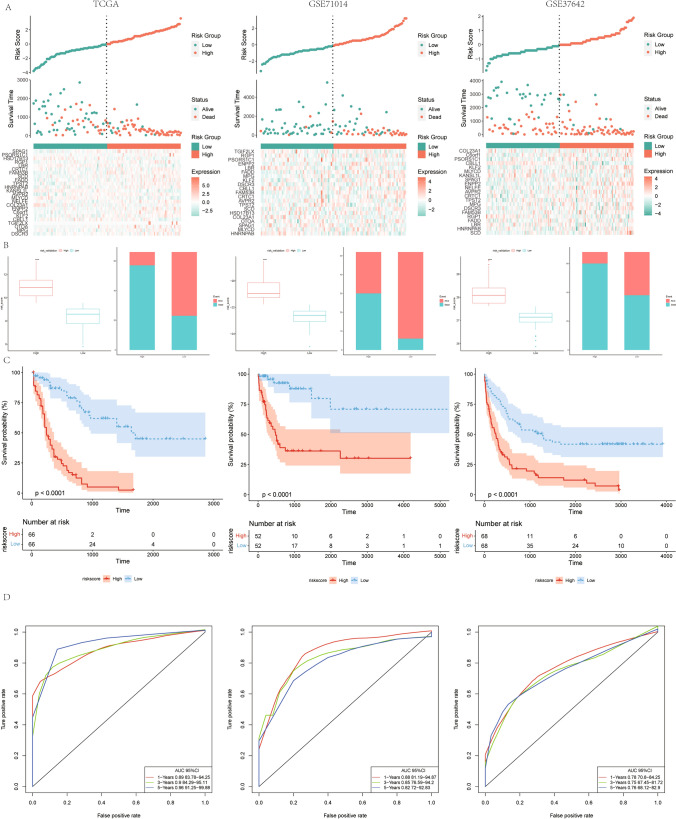
Prognostic performance of the TTKPI risk score model. **A**. Heatmap depicting gene expression, risk scores and survival times in high and low risk group samples, red represents death events and green represents survival events; **B**. Differences between high and low risk TTKPI risk groups in survival outcomes; **C**. Kaplan Meier survival analysis showing survival probability curve; **D**. Receiver operating curve analysis

The risk score of each tumor tissue sample was calculated based on the TTKPI risk score model, and the samples were divided into high and low risk score groups based on the median score (Fig. 6- A, B). Survival curve analysis for the training set data showed that the prognosis of the samples in the low-risk score group was better (p < 0.0001; Fig. 6C), and ROC analysis showed that the AUC of the samples reached 0.89, 0.9, and 0.96 at 1, 3, and 5 years, respectively, indicating that the model predicted well (Fig. 6D). In the independent dataset GSE71014, the prognosis was similarly significantly better in the low-risk score group (p < 0.0001; Fig. 6C). ROC analysis showed high AUC values at 1, 3, and 5 years was 0.88, 0.85, and 0.82 (Fig. 6D), respectively, indicating a good prognostic accuracy of the risk score model. Using the independent test dataset GSE37642, similar results showing significantly better prognosis of the samples in the low-risk score group (p < 0.0001; Fig. 6C), and the AUC values at 1, 3, and 5 years were 0.78, 0.75, and 0.76 (Fig. 6D), respectively, validating consistently high prognostic performance of the TTKPI risk scoring model.

### Methylation patterns and differentially methylated sites of the signature genes

**Fig. 7:**
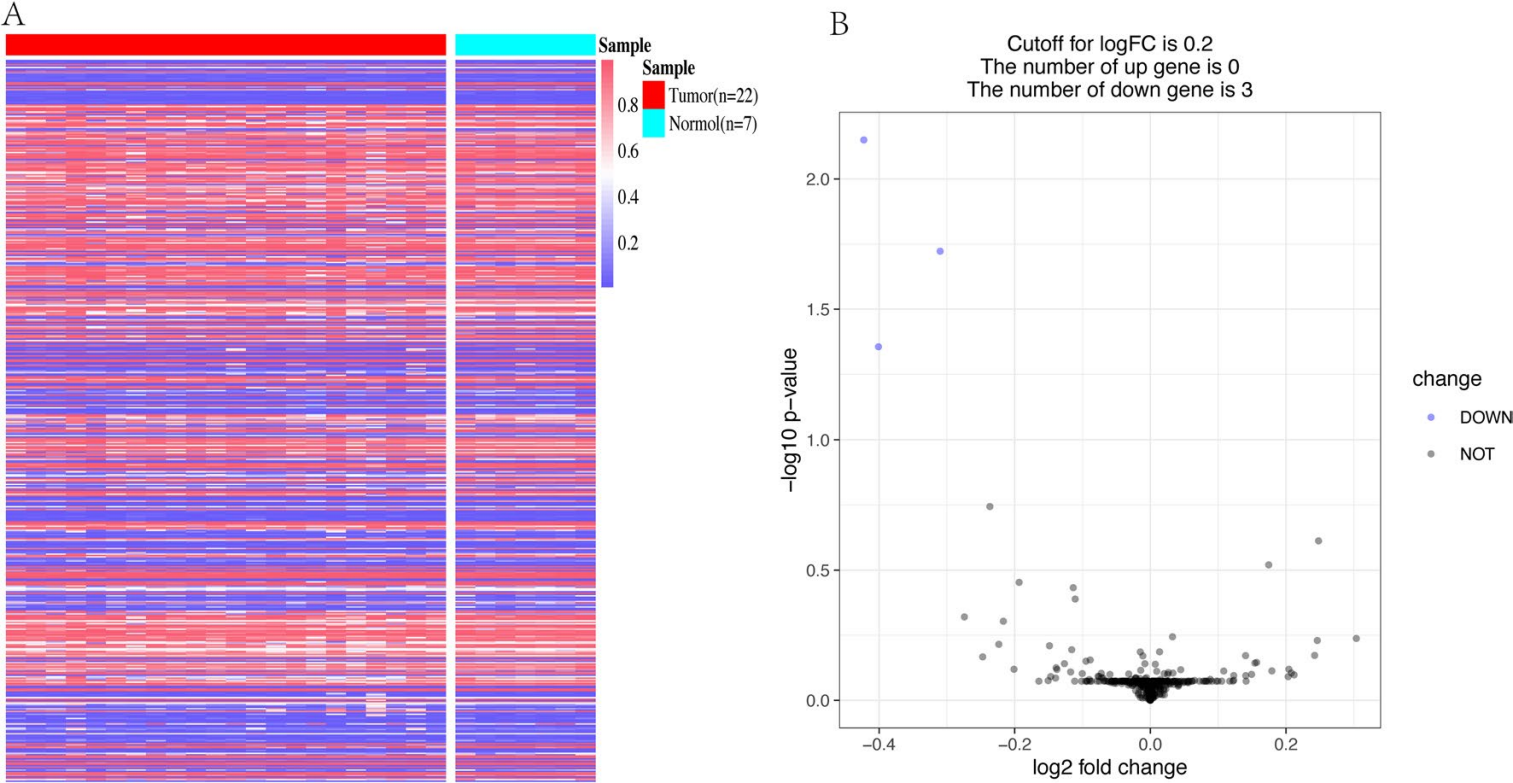
Methylation analysis of the signature genes in AML and normal samples. **A** Heatmap depicting the methylation site expression for the 20 signature genes that had available methylation data across 21 AML samples and 8 normal samples. **B** Volcano plot displaying the results of the differential expression analysis of the methylation sites between AML and normal samples

Figure 7A presents a heatmap depicting the expression of methylation sites corresponding to the 20 signature genes that had available methylation data. The heatmap visualizes the methylation patterns across the 21 disease samples and 8 normal samples. Figure 7B presents a volcano plot resulting from the differential expression analysis of the methylation sites, which helps to identify significantly differentially methylated sites between the disease and normal groups. Methylation sites that surpass both the adjusted P-value and logFC thresholds are considered significantly differentially methylated and are typically highlighted in the plot. The analysis identified three methylation sites (cg07545081, cg09924848, and cg04370247) corresponding to the FAM53B gene as differentially expressed. Overall, the methylation analysis of the signature genes revealed that the methylation sites of these genes were not significantly differentially expressed between the disease and normal groups.

### Mutation landscape of the signature genes in AML

**Fig. 8:**
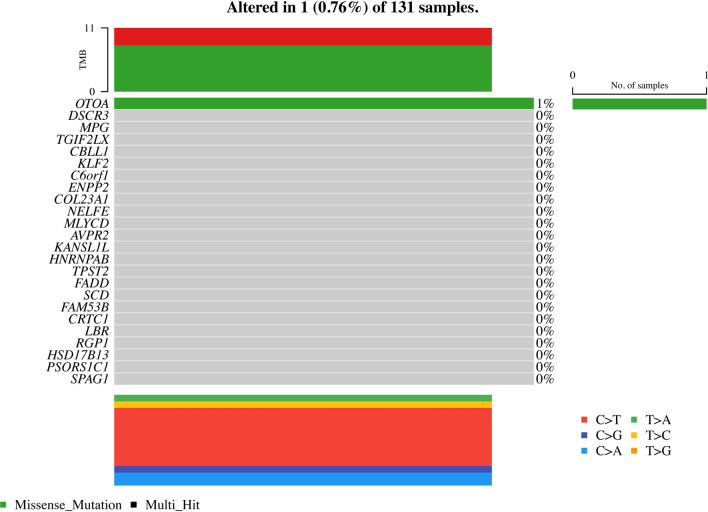
Mutation waterfall plot of the 24 signature genes in AML

The mutation analysis of the 24 signature genes in AML revealed that only three genes, namely OTOA, ENPP2, and FADD, harbored mutations in the analyzed samples (Fig. 8). The mutation waterfall plot provides a comprehensive overview of the mutation landscape of these genes across the AML samples. The plot highlights the presence of mutations in OTOA, ENPP2, and FADD, with the color intensity indicating the VAF of each mutation. The majority of the plot remains empty, reflecting the absence of mutations in the other signature genes. OTOA (Otoancorin) was found to have a notable missense mutation (p.T760M) with a variant allele frequency (VAF) of 0.3243 in one AML sample. ENPP2 (Ectonucleotide Pyrophosphatase/Phosphodiesterase 2) and FADD (Fas Associated Via Death Domain) were also identified to have mutations in the AML samples, although at lower frequencies compared to the OTOA mutation. Interestingly, the remaining 21 signature genes did not exhibit any mutations in the analyzed AML samples. This observation suggests that these genes may not be frequently mutated in AML or that the mutations in these genes were not detected in the specific cohort of samples investigated.

### TTKPI scores were associated with clinical phenotype

**Fig. 9:**
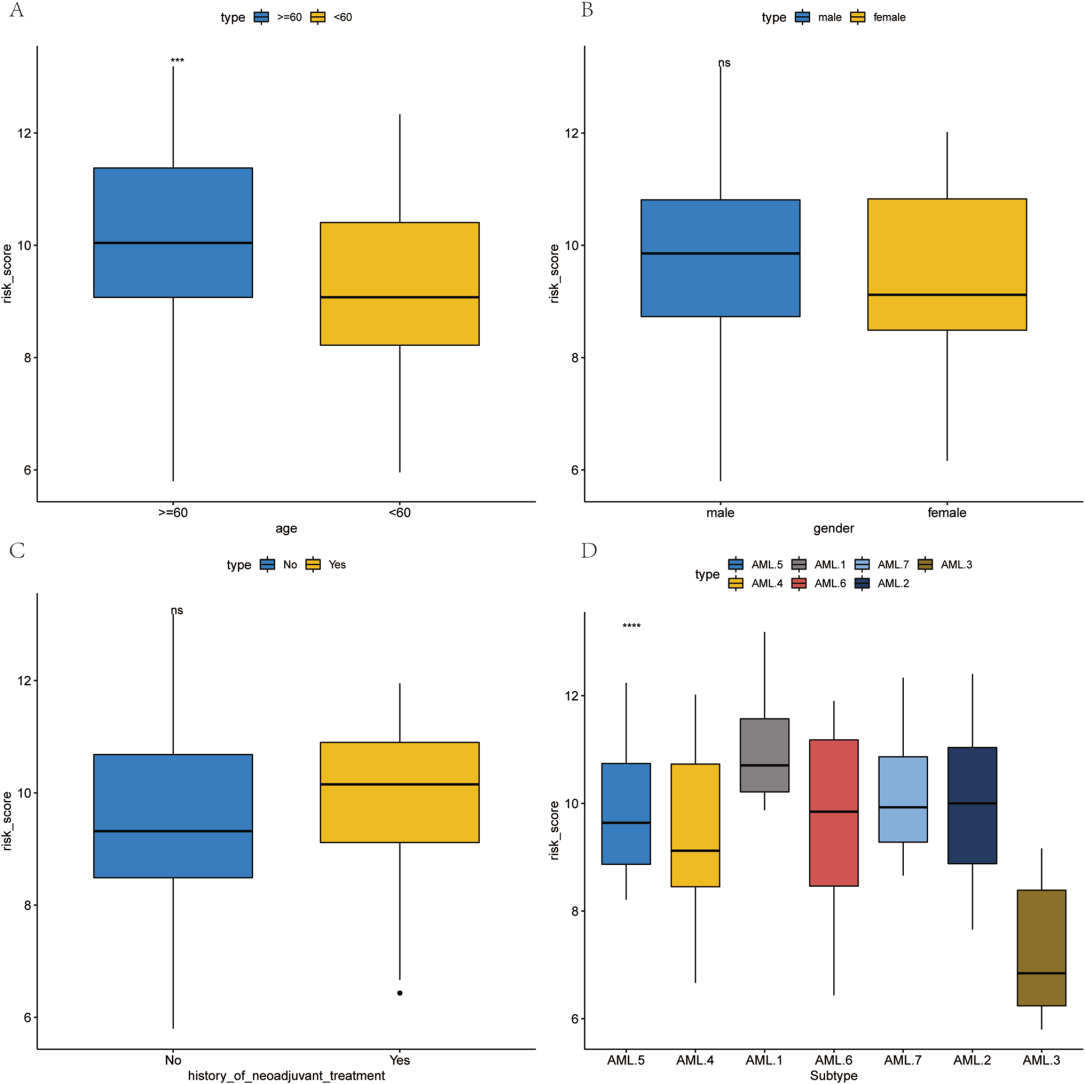
Intergroup comparison of TTKPI scores in clinical phenotype categories. (**A**) Comparison of TTKPI scores between age <60 and ≥60 groups. (**B**) Comparison of TTKPI scores between male and female groups. (**C**) Comparison of TTKPI scores between groups with and without history of neoadjuvant treatment. (**D**) Comparison of TTKPI scores among different AML subtypes

Figure 9 illustrates the comparison of TTKPI scores among different clinical phenotype categories. Figure 9A shows that the TTKPI risk score is significantly higher in the age ≥60 group compared to the age <60 (p < 0.0001). Figure 9B and 9C demonstrate no significant differences in TTKPI scores between male and female groups (p > 0.05) or between groups with and without a history of neoadjuvant treatment (p > 0.05), respectively. Figure 9D presents the comparison of TTKPI scores among different AML subtypes, revealing that the AML.1 subtype has significantly higher TTKPI risk scores than other subtypes (p < 0.00001), and the AML.3 subtype exhibits significantly lower TTKPI risk scores compared to other subtypes (p < 0.00001).

**Fig. 10:**
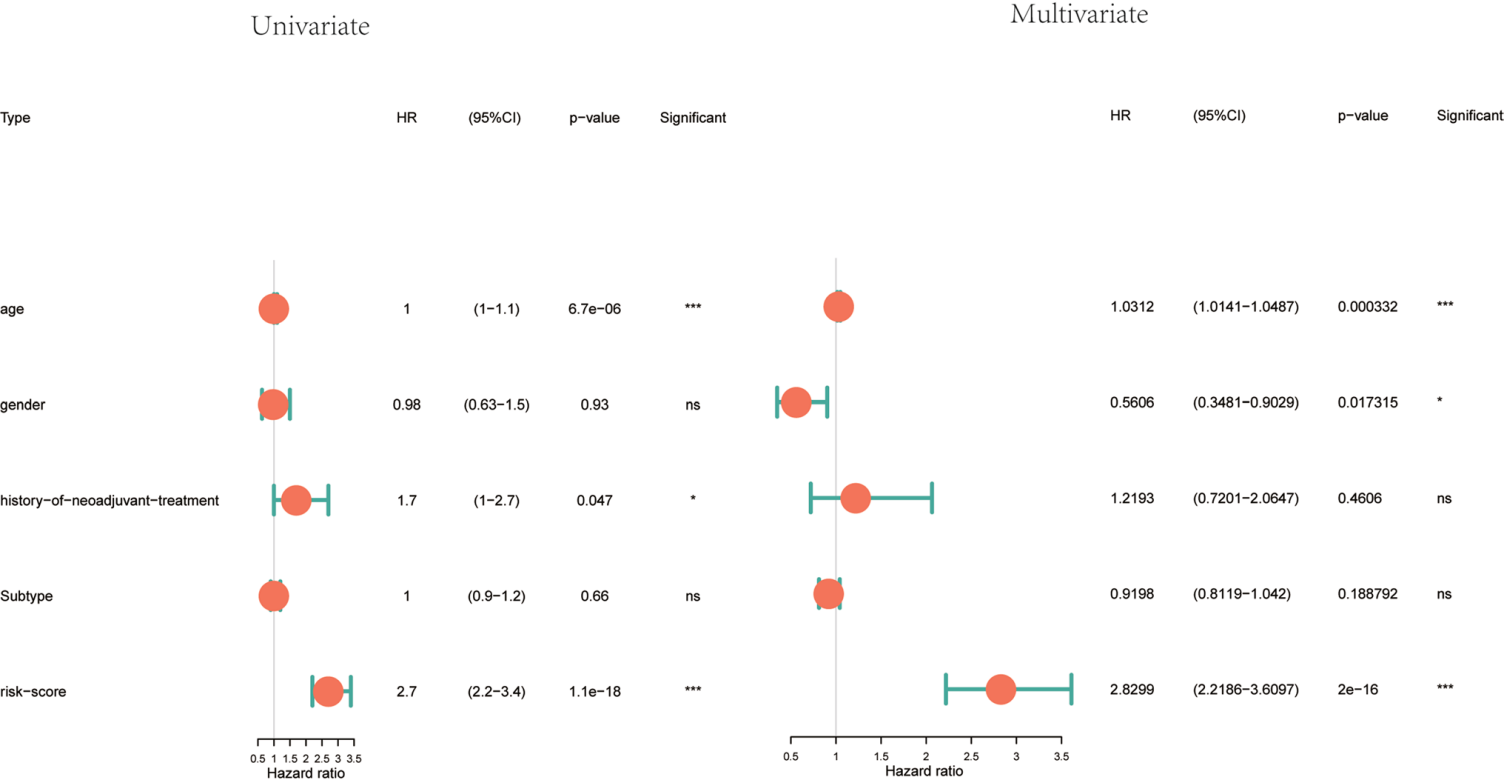
Forest plots of the results of single-factor COX and multi-factor COX analysis of clinical characteristics and TTKPI (ns:p > 0.05,*:p < 0.05,**:p < 0.01,***:p < 0.001,****:p < 0.0001)

The results of univariate and multivariate Cox regression analysis are depicted in forest plots (Fig. 10). The TTPKI risk score showed significantly elevated hazard ratio in both analyses.

**Fig. 11:**
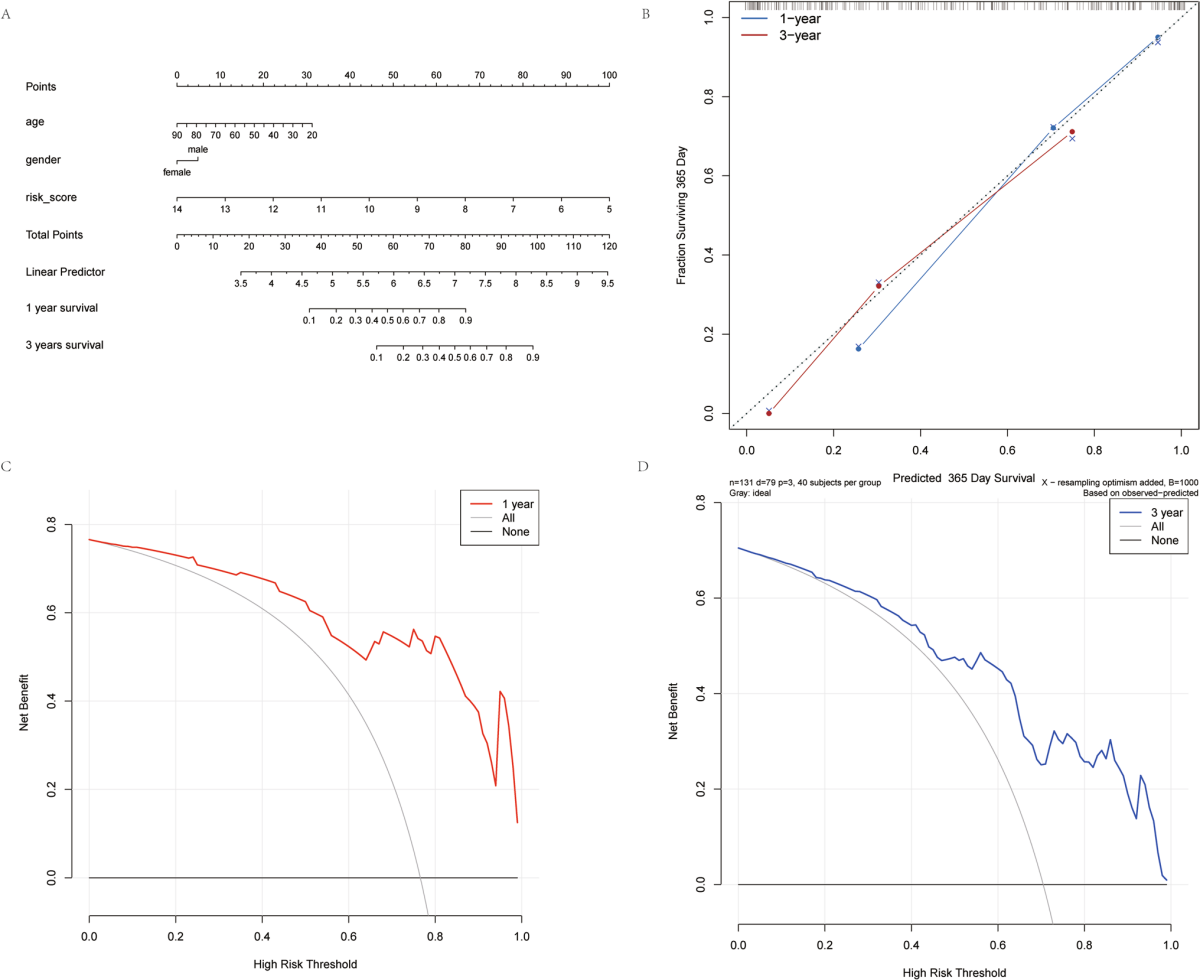
**A** shows the nomogram column line plot; **B** shows the calibration curve for the nomogram, with the 1 (blue line) and 3 (red line)-year survival periods, the dotted line represent perfect predictive performance; **C** and **D** show the decision curves for the 1 year and 3 year periods, the y-axis represents the net benefit, coloured line represents the nomogram (red: 1 year period, blue: 3 year period), grey line represents the assumption that all patients have adverse events, black line represents the assumption that no patients have adverse events

The result of the nomogram analysis is depicted in Fig. 11A and the calibration curves are shown in Fig. 11B. The two curves showed minimal deviation from the ideal prediction line, indicating that the combined model (column line plot) had high predictive performance for the 1-year and 3-year periods, and the two curves in the DCA decision curve (Fig. 11C, D) indicated that the column line plot constructed by the combined model showed better prognostic performance.

### TTKPI scores were associated with tumor immune cell infiltration.

**Fig. 12:**
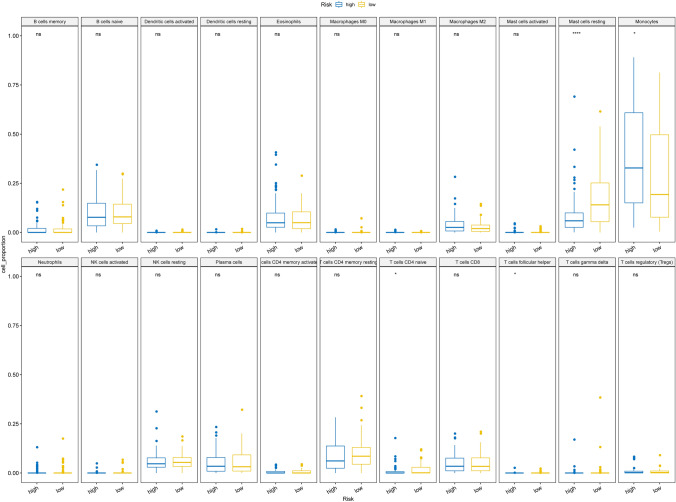
Differences in immune cell infiltration between high and low TTKPI subgroups. Differences in immune cell infiltration between high and low TTKPI subgroups. t-test *:p < 0.05,**:p < 0.01,***:p < 0.001,****:p < 0.0001

**Fig. 13:**
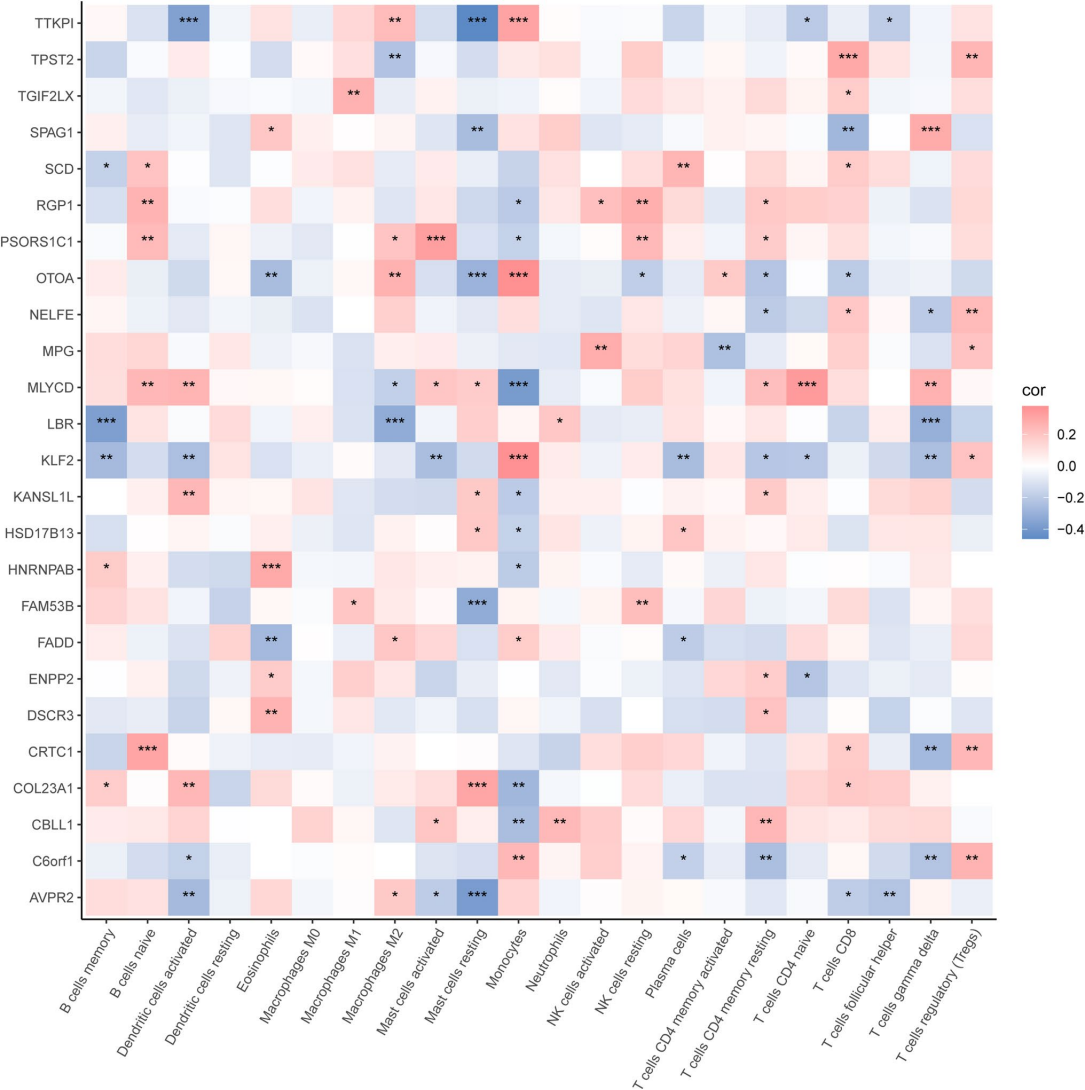
Correlation of TTKPI and its component genes with TME cells

CIBERSORT was used to analyse immune cell infiltration and determine differences between high and low TTKPI groups. The results showed that 4 types of infiltrating immune cells, “Mast cells resting”, “Monocytes”, “T cells CD4 naïve” and “T cells follicular helper”, were significantly different between the two groups (Fig. 12). The correlation between TTKPI scores, its constituent genes, and TME cells was calculated and “Dendritic cells activated”, “Mast cells resting”, “T cells CD4 naïve”, “T cells follicular helper” showed negative correlation with TTKPI, and T cells follicular helper showed negative correlation with TTKPI. “Macrophages M2”, “Monocytes” and other immune infiltrating cells showed positive correlation with TTKPI. (Fig. 13).

### TTKPI score-based prediction of immune checkpoint expression and immunotherapy responses

**Fig. 14:**
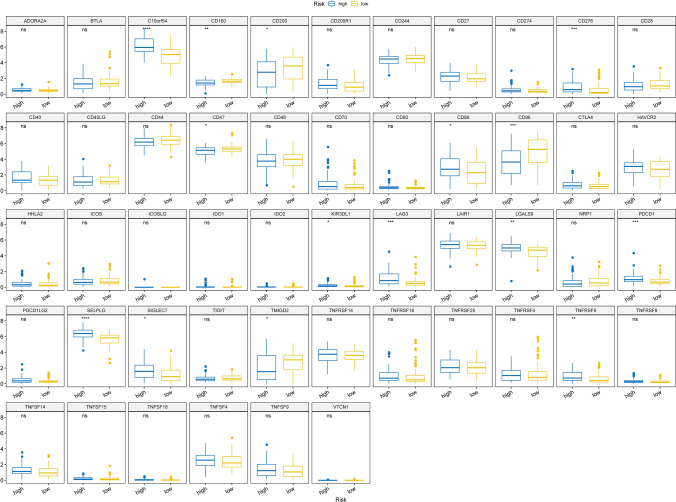
Differences in immune checkpoint expression between high and low TTKPI subgroups. Differences in immune checkpoint expression between high and low TTKPI subgroups. t-test *:p < 0.05,**:p < 0.01,***:p < 0.001,****:p < 0.0001

The differences in immune checkpoints between high and low TTKPI groups were analysed including C10orf54, CD160, CD200, CD276, CD47, CD86, CD96, KIR3DL1, LAG3, LGALS9, PDCD1, SELPLG, SIGLEC7, TMIGD2, TNFRSF8. The expression levels of CD160, CD200, CD47, CD96, TMIGD2 in the low-risk group were significantly higher than those in the high-risk group; the expression levels of C10orf54, CD276, CD86, KIR3DL1, LAG3, LGALS9, PDCD1, SELPLG, SIGLEC7, TNFRSF8 expression levels were significantly lower than those of the high-risk group. (Fig. 14).

### Molecular docking association TTKPI predicts potential therapeutic agents.

Screening of the corresponding compound structures from the DrugBank database according to Lipinski's rule yielded 5462 small molecule compounds. AVPR2, ENPP2, SCD and TPST2 were found to have corresponding spatial information structure, and the corresponding PDB files were downloaded: 7DW9, 5MHP, 4ZYO and 3AP1. DB11791, DB12886 and DB14773 were found to have good docking scores with the four proteins with Affinity < -9.6. The complete docking score table is shown in Table 4.

**Table 4:** Small molecule docking score with 4 proteins

DrugBank_ID	Name	Protein	Affinity (kcal/mol)
DB11791	Capmatinib	AVPR2	− 9.8
		ENPP2	− 11.2
		SCD	− 12.0
		TPST2	− 10.3
DB12886,	GSK-1521498	AVPR2	− 10.1
		ENPP2	− 12.7
		SCD	− 11.5
		TPST2	− 9.7
DB14773	Lifirafenib	AVPR2	− 9.9
		ENPP2	− 12.8
		SCD	− 13.1
		TPST2	− 10.8

**Fig. 15:**
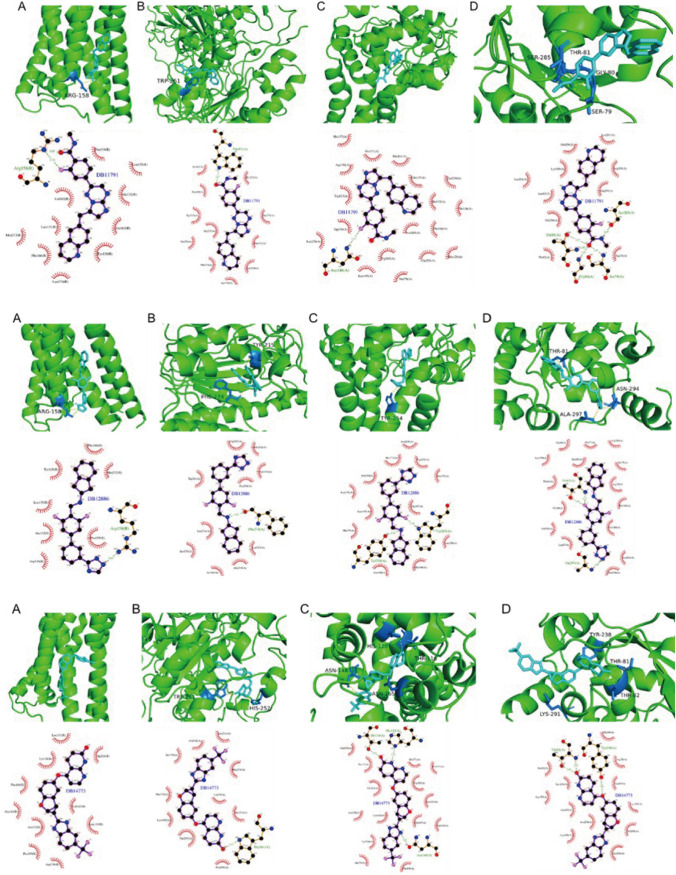
A Docking conformation and interaction force analysis of DB11791 (top), DB12886 (centre): DB14773 (bottom); with AVPR2 (**A**), ENPP2 (**B**), SCD (**C**) and TPST2 (**D**). Docking conformation and hydrogen bonding shown by Pymol (upper section), Ligplus interaction force analysis (lower section). Upper section: Pymol shows the docking conformation and hydrogen bonding, cyan represents the small molecule, yellow dashed line represents the hydrogen bond, blue represents the amino acid residues forming hydrogen bonds with the small molecule; lower section: Ligplus force analysis, the small molecule is seen the centre surrounded by the associated protein amino acid residues, green dashed line represents the hydrogen bonds formed, amino acid names for the amino acid residues forming hydrogen bonds are in green font

The docking conformation and interaction force analysis for AVPR2, ENPP2, SCD, and TPST2 with DB11791 (Fig. 15-top), DB12886 (Fig. 15-centre), DB14773 (Fig. 15-bottom) are represented.

## Discussion

The present study constructed and validated a T cell-mediated tumor killing sensitivity gene-based prognostic score, TTKPI, composed of 24 differentially expressed genes in AML. The TTKPI score showed good–excellent prognostic value for survival in the training and test datasets, with AUC values ranging from 75 to 96%. Higher TTKPI scores were associated with older age and cancer stage and the combined model showed better prognostic function. Distinct AML tumor immunological profiles clustering with age, T-cell receptor clonality and survival outcomes have been shown earlier [56]. The widely used ELN17 prognostic tool showed inadequate performance to predict long-term survival patients older than 60 years, despite accounting for mutational burden [57]. Older patients with AML show different memory T cell subpopulations that young populations, owing to T cell senescence and terminal differentiation, particularly in case of CD8 + T cells [58]. Together, these evidences support the application of combined prognostic tools integrating tumor immunological scores such as TTKPI with clinical parameters for improved prognostic performance.

Our TTKPI prognostic scoring system incorporates 24 differentially expressed T-cell-mediated tumor-killing sensitivity genes, several of which have been shown to play critical roles in the pathogenesis of AML. For instance, Sperm-associated antigen 1 (SPAG1) is widely expressed in acute myeloid leukemia (AML) patients and is associated with a poor prognosis [59]. It promotes AML cell proliferation and survival by activating the ERK/MAPK signaling pathway and affects AML cell susceptibility to venetoclax, a chemotherapy drug [60]. Taken an example of HNRNPAB, clinical studies have consistently shown that elevated HNRNPAB expression in AML patients is associated with adverse outcomes [61]. It plays a role in AML pathogenesis by interacting with the key hematopoietic transcription factors CEBPA (CCAAT Enhancer Binding Protein Alpha) and SPI1 (Spleen Focus Forming Virus (SFFV) and influencing leukemogenesis [62]. HNRNPAB’s involvement in splicing decisions may contribute to leukemogenesis by generating isoforms that promote cell survival, proliferation, or drug resistance [62]. For another instance, KLF2 acts as a tumor suppressor in AML, and its upregulation promotes apoptosis and differentiation of AML cells [63]. KLF2 is a downstream effector of the AMPK signaling pathway, mediating AMPK activation-induced death receptor pathway-dependent apoptosis and myeloid differentiation in AML cells [63].

A high TTKPI score predicted worse survival with good accuracy. T cell mediated tumor killing sensitivity genes have shown strong prognostic value in several solid tumor types including lung adenocarcinoma, head and neck cancer and hepatocellular carcinoma [48, 64, 65]. AML is highly heterogenous at the molecular level with low mutational burden, which is believed to underpin modest responses to antibody dependent checkpoint inhibitor therapy as compared to solid tumors and receptor independent T-cell directed therapies assumes high significance [66]. Identifying GSTTK based tumor immunological subtypes can also be very valuable for predicting responses to checkpoint inhibitors and emerging immunotherapies such as CAR T cell and bispecific antibody therapy. Our data showed different patterns of checkpoint expression in the high and low TTKPI groups. The low TTKPI group showed higher levels of CD160, CD200, CD47, CD96, TMIGD2, and lower levels of C10orf54, CD276, CD86, KIR3DL1, LAG3, LGALS9, PDCD1, SELPLG, SIGLEC7, TNFRSF8. Classical immune checkpoint inhibitor drugs targeting PD-1, PD-L1 and CTLA4 show variable efficacy and additional checkpoint inhibitor drugs such as drugs targeting LAG3 are under current focus [67, 68]. Our data suggest that the TTKPI score pattern could potentially direct the selection of optimal checkpoint inhibitor drugs and combination. The TTKPI score component GSTTKs were enriched in immune-related pathways including antigen processing and presentation, T cell extravasation, interleukin-1-mediated signalling pathway; key pathways of tumor immune function, which maps the TTKPI score to functional variances in the tumor microenvironment. TTKPI negatively correlated with CD4 naïve and follicular helper T cell immune infiltration scores while M2 macrophages, monocytes were positively correlated, implying a T cell exhausted, comparatively pro-inflammatory, innate immune dominated milieu in high TTKPI states, which corresponded to low survival. T cell exhaustion and concurrent PD-1 resistance is a characteristic of severe AML [67, 68]. An AML tumor immune landscape marked by high M2 macrophage and monocyte infiltration corresponds to high inflammatory response and predicts poor survival [69], in alignment with our results.

Several studies have investigated the prognostic value of gene signatures in AML. For example, Stanley et al. developed a 17-gene leukemia stem cell (LSC) score based on differential expression between LSC + and LSC- cell fractions, which serves as a powerful prognostic tool to rapidly identify AML patients with poor outcomes who may benefit from alternative treatment strategies [70]. Docking et al. [71] used least absolute shrinkage and selection operator (LASSO) regression to derive a 16-gene expression signature, which could be used to improve risk stratification for a broad spectrum of AMLs [71]. Zhu et al. [72] developed a novel 6 immune-related gene (IRG) signature prognostic model for AML patients based on immunogenomic landscape analysis, which could effectively stratify AML patients into high and low risk groups and serve as an independent prognostic factor [72]. Lu et al. [73] developed a novel CXC chemokine receptors (CXCRs) gene signature-based risk model that can effectively stratify AML patients and serve as an independent prognostic factor [73]. Jiang et al. [74] developed a novel hypoxia-related gene signature prognostic model for AML patients, which can effectively predict clinical outcomes, reflect the immune microenvironment response intensity, and serve as an independent prognostic indicator for AML [74]. However, these studies did not specifically focus on T cell-mediated tumor killing sensitivity genes (GSTTK) or the comprehensive analysis of the immune microenvironment. Our study stands out in its focus on GSTTK and the development of a comprehensive TTKPI that captures the functional state of T cells in the AML microenvironment. By investigating the expression patterns of immune checkpoints and T cell infiltration in the context of the TTKPI, we provided novel insights into the immunological landscape of AML and its potential implications for precision immunotherapy. Methodologically, our study employed a rigorous bioinformatic approach, integrating multiple datasets for training and validation, and utilizing both Cox regression and LASSO regression to identify the most informative genes for the TTKPI score. Furthermore, we explored the biological relevance of the TTKPI component genes through functional enrichment analyses and examine their associations with clinical characteristics, immune cell infiltration, and immune checkpoint expression. While previous studies have investigated prognostic gene signatures and immune-related factors in AML, our study provided a unique contribution by focusing specifically on T cell-mediated tumor killing sensitivity genes and developing a comprehensive prognostic index that captures the functional state of T cells in the AML microenvironment. The novelty of our approach lies in the integration of T cell-specific gene signatures, immune checkpoint expression, and immune cell infiltration to provide a more holistic view of the immunological landscape in AML and its potential for guiding precision immunotherapy.

Our findings may have implications for immunotherapy selection based on TTKPI profiles. The TTKPI score pattern could potentially direct the selection of optimal checkpoint inhibitor drugs and combination. TTKPI prognostic model can not only predict survival, but also capture functional differences in the tumor immune microenvironment, and is expected to guide the selection of biomarker-based precision immunotherapy regimens. Our study provides a basis for further clinical studies to validate the application value of TTKPI scores in prognosis and precision medication of immunotherapy. In addition, the strengths and limitations of this research should be highlighted. The strengths of the present study include a dual step selection for identification of a GSTTK genes for prognostic score construction and validation in an independent cohort. In addition, tumor immune cell infiltration analysis, checkpoint expression patterns and molecular docking were leveraged to shed light on functional and therapeutic implications of the constructed TTKPI score. The limitations of the present study include a small number of validation datasets and the lack of experimental studies to validate the GSTTK signature and its functional aspects identified in the in-silico study. Overall, our findings demonstrated the prognostic value of the TTKPI score and identified functional pathways, molecular targets, and potential agents that may be harnessed for tumor killing T-cell dependent therapies in AML.

Several avenues for future research that require experimental validation are proposed to further explore the biological underpinnings and clinical implications of the TTKPI score. Firstly, in vitro functional assays using AML cell lines and primary samples could be performed to validate the role of TTKPI component genes in T cell-mediated tumor killing. These experiments may include knockdown or overexpression of TTKPI genes, co-culture assays with T cells, and cytokine profiling. Secondly, in vivo animal models, such as xenograft or humanized mouse models, could be employed to assess the impact of TTKPI gene modulation on tumor growth, survival, and antitumor immunity. Thirdly, prospective clinical studies in AML patients should be designed to confirm the clinical utility of the TTKPI score, including multicenter validation, longitudinal studies to predict immunotherapy response, and integration with other risk factors for personalized management. Lastly, mechanistic studies using single-cell RNA sequencing, epigenetic profiling, and proteomic/metabolomic analyses could help elucidate the biological mechanisms underlying the TTKPI score. By conducting these comprehensive experiments, future studies can provide a more in-depth understanding of the TTKPI score's biological significance and guide the development of novel immunotherapeutic strategies and risk-adapted management approaches for AML patients.

Several recommendations for future research are worthwhile to be mentioned. Firstly, prospective validation of the TTKPI score in larger, independent cohorts of AML patients from diverse backgrounds is essential to confirm its prognostic value and clinical utility. Secondly, integrating the TTKPI score with other established prognostic factors, such as cytogenetic and molecular markers, may help refine risk stratification and treatment decision-making. Thirdly, the TTKPI score could guide the selection of patients for novel immunotherapeutic approaches, with prospective clinical trials stratifying patients based on their TTKPI scores to identify subgroups most likely to benefit from specific immunotherapies. Furthermore, the key genes identified in the TTKPI score warrant further functional characterization to elucidate their roles in AML pathogenesis and potential as therapeutic targets. Lastly, exploring novel biomarkers that capture additional aspects of the immune microenvironment in AML, such as cytokine profiles or single-cell transcriptomic data, may provide a more comprehensive understanding of the immune landscape and identify new prognostic and predictive biomarkers. We believe that pursuing these lines of investigation will contribute to better patient outcomes and advance our understanding of the complex interplay between the immune system and leukemia biology.

## Conclusion

The present study identified a 24 T-cell mediated tumor killing sensitivity gene signature of AML and constructed a comprehensive risk score TTKPI, which showed good–excellent prognostic value for overall survival in AML and was significantly associated with clinical phenotype features. Immune cell infiltration analysis showed that the TTKPI score corresponded to the tumor microenvironment and immune checkpoint expression patterns, and using drug docking, we identified 3 small molecule drugs with high potential for clinical translation. The utility of TTKPI score for prognosis and targeted biomarker-based immunotherapy should be tested in clinical studies.

## Limitations section

(1) The study relied on publicly available datasets, which might have inherent biases or limitations in terms of patient selection, data quality, or clinical annotation. Future studies using prospectively collected, well-characterized cohorts could help validate and extend the findings. (2) The prognostic performance of the TTKPI score was evaluated using retrospective data. Prospective validation in additional independent cohorts is necessary to establish its clinical utility and applicability in real-world settings. (3) The study focused on the transcriptomic landscape of AML and did not incorporate other omics data, such as genomics, epigenomics, or proteomics. Integration of multi-omics data could provide a more comprehensive understanding of the molecular mechanisms underlying the TTKPI score and its prognostic significance. (4) The functional implications of the TTKPI score and its component genes were primarily inferred from bioinformatic analyses. Experimental validation using in vitro or in vivo models is needed to confirm the biological roles and therapeutic potential of the identified genes and pathways. (5) The study did not explore the potential impact of different treatment regimens or specific targeted therapies on the prognostic value of the TTKPI score. Future research could investigate how the TTKPI score performs in the context of specific treatment modalities and whether it can guide personalized treatment decisions.

### Supplementary Information

**Additional file 1.** The version of all of the R packages that this study has utilized.

**Additional file 2.** The Kaplan Meier plot of 24 key prognostic genes.

## Data Availability

The two datasets were de-batched and combined into the training set data. GSE71014 and GSE37642 datasets were downloaded from GEO (https://www.ncbi.nlm.nih.gov/geo/). All data generated or analysed during this study are included in this published article.
